# Transcriptome and metabolome analysis of flavonol synthesis in apricot fruits

**DOI:** 10.3389/fpls.2023.1187551

**Published:** 2023-06-14

**Authors:** Xueping Han, Jinzheng Wang, Guiping Wang, Fang Dong, Peixian Nie, Xiaomin Xue

**Affiliations:** Shandong Institute of Pomology, Shandong Academy of Agricultural Sciences, Taian, China

**Keywords:** Chinese apricot, metabolomics, RNA-Seq, flavonols, Flesh

## Abstract

**Introduction:**

Apricot fruits are edible and serve as a source of medicinal compounds. Flavonols are important plant secondary metabolites that have antioxidant and antitumor effects and may promote cardiovascular health.

**Methods:**

The flavonoid content in three stages of the ‘Kuijin’ and the ‘Katy’ was observed, followed by the combination of metabolome and transcriptome analysis to explore the metabolic basis of flavonol synthesis.

**Results:**

The differences in the metabolite contents between stages (of the same cultivar) and between cultivars (at the same stage) revealed decreases in the flavonoid content as fruits developed (i.e., from 0.28 mg/g to 0.12 mg/g in ‘Kuijin’ and from 0.23 mg/g to 0.05 mg/g in ‘Katy’). To decipher the regulation of flavonol synthesis in apricot (Prunus armeniaca L.), the metabolomes and transcriptomes of fruit pulp at three developmental stages of ‘Kuijin’ and the ‘Katy’ were analyzed. A total of 572 metabolites were detected in ‘Kuijin’ and the ‘Katy’ pulp, including 111 flavonoids. The higher flavonol content young ‘Kuijin’ fruits at 42 days after full bloom is mainly due to 10 types of flavonols. Three pairs of significant differences in flavonol content were identified. From these three comparison groups, three structural genes were strongly correlated with the levels of 10 types of flavonols (Pearson correlation coefficients > 0.8, p value < 0.05), including PARG09190, PARG15135, and PARG17939. The weighted gene co-expression network analysis showed that the turquoise module genes were highly correlated with flavonol contents (P < 0.01). There were 4897 genes in this module. Out of 4897 genes, 28 transcription factors are associated with 3 structural genes based on weight value. Two of the transcription factors are not only associated with PARG09190 but also with PARG15135, indicating their critical importance in the flavonols biosynthesis. The two TFs are PARG27864 and PARG10875.

**Discussion:**

These findings provide new insights into the biosynthesis of flavonols and may explain the significant differences in flavonoid content between the ‘Kuijin’ and the ‘Katy’ cultivars. Moreover, it will aid in genetic improvement to enhance the nutritional and health value of apricots.

## Introduction

Apricot (*Prunus armeniaca* L.), which is a stone fruit species belonging to the subgenus *Prunophora* of the genus *Prunus* in the subfamily Prunoidae of the family Rosaceae, originated in China and has been cultivated for almost 3,000 years (Rieger, 2006). Edible apricot fruits are considered to be a functional food with nutritional and bioactive properties (García-Gómez et al., 2020). They are rich in nutritionally valuable flavonoids and contain relatively large amounts of vitamin C, polyphenols, carotenoids, and other antioxidant compounds (Dragovic-Uzelac et al., 2005; Fan et al., 2018; Fratianni et al., 2018). Flavonoids are major plant secondary metabolites that are crucial for biological processes related to plant growth (Krueger, 2007). Flavonols are the most abundant and widely distributed flavonoids, including quercetin, kaempferol, myricetin, rhamnetin, morin, fisetin, galangin, azaleatin, and their respective glycosyl derivatives (Longbo et al., 2016). Specifically, they possess antioxidant, antitumor, and anti-inflammatory properties and protect the nervous system, while also helping to prevent cardiovascular diseases and diabetes (Wang et al., 2008; Perez-Vizcaino and Duarte, 2010; Dajas, 2012; Valentová et al., 2014; Devi et al., 2015). Although flavonols have been studied in several fruits, including peaches *(Prunus persica* L. Batsch) (Cao et al., 2019), grapes (*Vitis vinifera*)(Castillo-Muñoz et al., 2007), strawberries (*Fragaria × ananassa*) (Griesser et al., 2008), loquats (*Eriobotrya japonica Lindl*) (Zhang et al., 2015), and blueberries (*Vaccinium corymbosum*)(Colle et al., 2019; Wang and Vodovotz, 2019). Apricot is a very useful species for identifying key genes involved in fruit development and ripening (García-Gómez et al, 2021). There has been limited research on the flavonols in the apricot fruit pulp.

‘Katy’ and ‘Kuijin’ are the two main apricot cultivars grown in Shandong province. ‘Kuijin’ contains more abundant flavonoids compared to ‘Katy’. Studying the biosynthesis mechanism of flavonoid compounds will help breed apricot cultivars with high flavonoid content. Observing changes in the transcriptome and metabolome of developing ‘Katy’ and ‘Kuijin’ fruits can help elucidate the regulatory mechanisms responsible for high flavonol contents and breeding cultivars that produce fruits with a desirable bioactive flavonol conten.

## Materials and methods

### Plant materials

**Figure 1:**
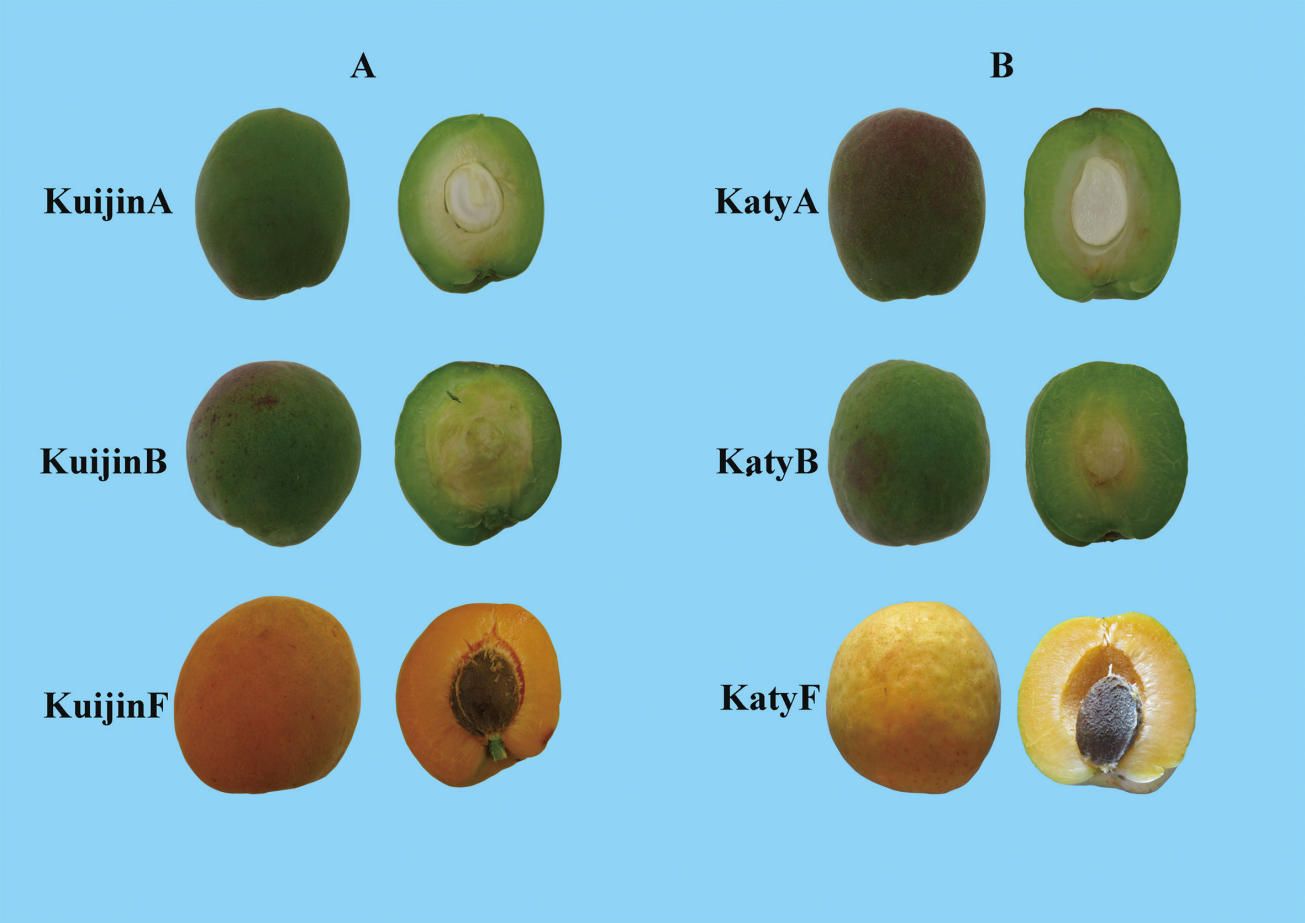
Phenotypes of apricot cultivars ‘Katy’ and ‘Kuijin’ during different periods. **(A)**, young fruit period; **(B)**, color-changing period; F, matureperiod.

‘Katy’ and ‘Kuijin’ apricot fruits were obtained from the Taidong experimental orchard of the Shandong Institute of Pomology (Taian, Shandong, China). Uniformly developing fruits at the same stage were collected from three disease- and insect-free per cultivar. Samples were collected during the young fruit period (42 days after full bloom; A), the color-changing period (52 days after full bloom; B), and the mature period (84 days after full bloom; F) (**Figure 1**). Thus, six samples (KatyA, KatyB, KatyF, KuijinA, KuijinB, and KuijinF) with three biological replicates each (18 samples in total) were collected in 2018. The collected fruits were immediately frozen in liquid nitrogen and stored at −80°C until analyzed.

### Metabolite extraction and identification

Freeze-dried pulp samples were crushed using zirconia beads and the MM 400 mixer mill (30 Hz for 1.5 min) (Retsch, Haan, Germany). The resulting powder was weighed and 100 mg was mixed with 1.0 mL 70% aqueous methanol and then incubated overnight at 4°C. After centrifuging the sample at 10,000 g for 10 min, thesupernatant was added to the CNWBOND Carbon-GCB SPE Cartridge (250 mg, 3 mL; ANPEL, Shanghai, China) and filtered (SCAA-104, 0.22 μm pores; ANPEL). The metabolites were quantified using a triple quadrupole mass spectrometry system in the multiple reaction monitoring mode and then identified using the MetWare database and other publicly available metabolite databases. The Analyst (v1.6.1) software (AB SCIEX, ON, Canada) was used to process the mass spectrometry data. After obtaining the mass spectra of the metabolites in different samples, the peak areas of all mass spectral peaks were integrated and the mass spectral peaks of the same metabolite in different samples were integrated and corrected (Fraga et al., 2010). Furthermore, a principal component analysis (PCA) was performed to analyze the metabolite data. A partial least squares discriminant analysis (PLS-DA) was completed to maximize the differences in metabolites between groups and identify the metabolites. The variable importance in projection (VIP) value of the OPLS-DA model was combined with the P-value or fold-change data in the univariate analysis to screen for differentially metabolites. Metabolites with a VIP ≥1 and a fold-change ≥2 or ≤0.5 were considered to be significantly different.

### RNA extraction, transcriptome sequencing, and *De Novo* assembly

TRIzol reagent (Invitrogen, China) was used to extract total RNA from the pulp of ‘Kuijin’ and ‘Katy’ apricot fruits collected at three developmental stages (A, B, and F). The integrity of the extracted RNA and the presence of contaminating DNA were analyzed by agarose gel electrophoresis and the NanoPhotometer system (Implen GmbH, Munich, Germany). The 2100 Bioanalyzer (Agilent Technologies, Santa Clara, CA, USA) was used to verify the purity and quantity of the RNA. Triplicate samples of pulp at each developmental stage were used to construct 18 apricot transcriptome libraries, which were sequenced using the Illumina HiSeq™ 2000 system at the Beijing Genomics Institute to produce 150-bp paired-end reads. Raw sequencing reads were filtered by removing reads containing adapters or more than 10% unknown nucleotides (N) and low-quality reads [i.e., more than 50% low quality (Q <20) bases] using FastQC (http://www.bioinformatics.babraham.ac.uk/projects/fastqc/ ). The clean reads were mapped to the apricot reference genome (https://www.ncbi.nlm.nih.gov/genome/?term=apricot ) using HISAT2 (v2.0.5) (Kim et al., 2013).

### Transcriptome data analysis

Gene expression levels were calculated on the basis of the fragments per kilobase of transcript per million fragments mapped (FPKM) value. Genes that were differentially expressed between two samples (KatyA *vs* KatyB, KatyA *vs* KatyF, KatyB *vs* KatyF, KuijinA *vs* KuijinB, KuijinA *vs* KuijinF, KuijinB *vs* KuijinF, KuijinA *vs* KatyA, KuijinB *vs* KatyB, and KuijinF *vs* KatyF) were analyzed using the DEGseq R package (Love et al., 2014). The non-standardized raw read count data for the genes were used, and the read count for the genes was determined using featureCounts (Li, 2014). After the differential gene expression analysis, the hypothesis test P-value was corrected according to the Benjamini–Hochberg method to obtain the false discovery rate (FDR). Genes with |log_2_(fold-change)| ≥1 and FDR <0.05 were screened as DEGs, which were subsequently functionally annotated *via* Gene Ontology (GO) and Kyoto Encyclopedia of Genes and Genomes (KEGG; https://www.genome.jp/kegg) pathway analyses (Yonekura et al., 2012).

### Weighted gene co-expression network analysis and gene regulatory network visualization

Co-expression networks were constructed using the WGCNA (v1.29) package in R (Langfelder and Horvath, 2008). Among the 12593 genes, those with an average FPKM >1 (from three replicates) were used for the WGCNA. The modules were obtained using the automatic network construction function blockwise. The default settings were applied, with the exception of the soft power (18), the min module size (50), and the merge cut height (0.45). The eigengene value was calculated for each module and used to test the associations with the metabolism of flavor-related compounds.

### Real-time quantitative PCR

Total RNA was extracted from the samples using the HiPure HP Plant RNA Mini Kit (Magen R4165-02) and then reverse transcribed to cDNA using the SUM Onetube RT Mixture III (gDNA removal) (SUMMER SUM7806a). On the basis of the gene names, the corresponding sequences were obtained and used to design primers. The relative expression levels of 12 candidate genes were determined by qPCR, which was completed using the CFX 96 Real-time PCR assay system (Bio-Rad, Hercules, CA, USA). The SYBR Green dye method was used, and *PaACIIN* was the internal reference gene. Seven structural genes and five transcription factor genes were candidate genes. The relative gene expression levels were calculated according to the 2^−ΔΔCt^ method. The qPCR program was as follows: 94°C for 10 min; 40 cycles of 94°C for 10 s, 60°C for 30 s, and 55°C for 30 s. The qPCR analysis was conducted using three biological replicates. Details regarding the qPCR primers are listed in **Table S8**.

### Statistical analysis

The SPSS 22.0 software (SPSS Inc., Chicago, IL, USA) was used for the one-way ANOVA with a Tukey test. The threshold for determining significant differences was P ≤ 0.05. Data are provided herein as the mean ± standard deviation.

## Results

### Metabolites in ‘Katy’ and ‘Kuijin’ apricot fruits at three developmental stages

**Figure 2:**
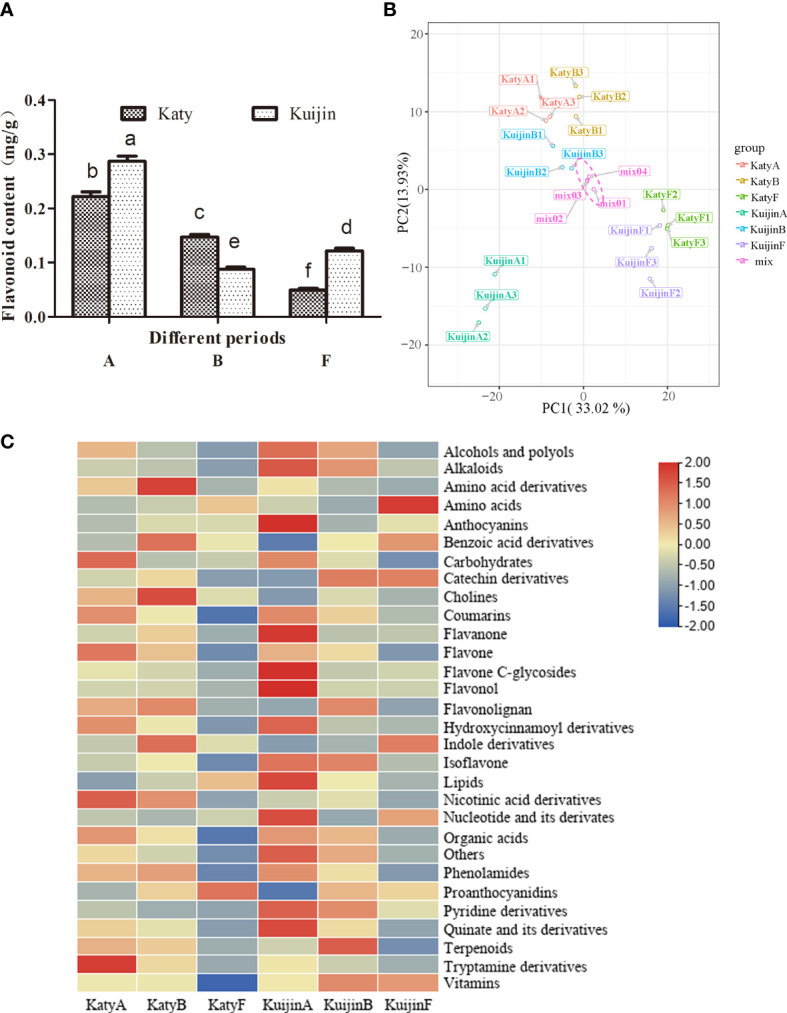
Global changes in the metabolite profiles of ‘Kuijin ‘ and ‘Katy ‘ fruits in three developmental stages. **(A)** Flavonoid contents in ‘Kuijin ‘ and ‘Katy ‘ fruits. Data are presented as the mean ± standard deviation (n = 3). Significance was assessed using P ≤ 0.05 as the threshold. **(B)** Principal component analysis of KuijinA, KuijinB, KuijinF, KatyA, KatyB, and KatyF. **(C)** Heat map clustering of metabolites by TBtools. The significance was assessed using P ≤ 0.05 as the threshold, and lowercase letters a, b, c, d, e, f were used to represent it.

To analyze the changes in the flavonoid content of apricot fruits at different developmental stages, the total flavonoid contents of ‘Katy’ and ‘Kuijin’ fruits collected in three periods were measured (**Figure 1**). The flavonoid content decreased from 0.28 mg/g to 0.12 mg/g during the development of ‘Kuijin’ fruits, whereas it decreased from 0.23 mg/g to 0.05 mg/g during the development of ‘Katy’ fruits. The total flavonoid content was significantly higher in the ‘KuijinA’ samples than in the other samples (**Figure 2A**). The changes in the flavonoid metabolites in ‘KuijinA’ were investigated further (**Table S1**). The PCA results indicated that the biological replicates had similar metabolite profiles, with KuijinA significantly separated from KuijinB, KuijinF, KatyA, KatyB, and KatyF along the principal component 1 (PC1) axis and the PC2 axis (**Figure 2B**, **Figure S1**). A total of 572 metabolites were identified in the ‘Katy’ and ‘Kuijin’ fruits at three developmental stages (**Table S2**). These metabolites, which were divided into 30 groups, included 111 flavonoids (12 catechin derivatives, 16 flavanones, 25 flavones, 9 flavone C-glycosides, 29 flavonols, 1 flavonolignan, 7 isoflavones, 7 anthocyanins, and 5 proanthocyanidins) (**Figure 2C**). Upon comparing the classification of flavonoid compounds, it was found that the levels of flavanol, flavanone, and flavonec-glycosides were significantly higher during the ‘KuijinA’ compared to other stages. Therefore, a further comparison was made to analyze the types and contents of flavonoid compounds during each stage.

### Flavonols In ‘Katy’ and ‘Kuijin’ apricot fruits at three developmental stages

**Figure 3:**
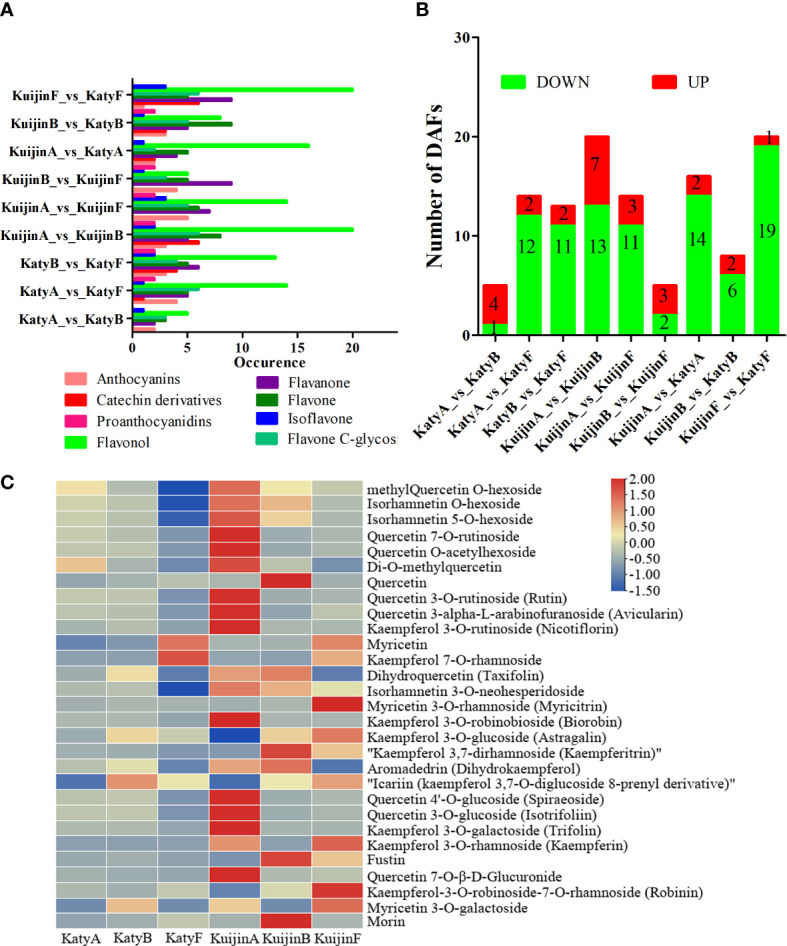
Liquid chromatography electrospray ionization tandem mass spectrometry analysis of the flavonol types and relative contents. **(A)** Comparison of flavonoid types in different samples. **(B)** Number of differentially accumulated flavonols (DAFs) among the comparisons. **(C)** Relative contents of differentially accumulated flavonols in ‘Katy’ and ‘Kuijin’ fruits at three developmental stages as determined using TBtools.

After comparing the flavonoid compounds, it was observed that 16, 38, 39, 52, 42, 30, 32, 36, and 50 compounds were differentially accumulated in the comparisons of KatyA vs KatyB, KatyA vs KatyF, KatyB vs KatyF, KuijinA vs KuijinB, KuijinA vs KuijinF, KuijinB vs KuijinF, KuijinA vs KatyA, KuijinB vs KatyB, and KuijinF vs KatyF, respectively as shown in (**Figure 3A**). The result indicates that the group with the highest number of accumulated flavonoid compounds is KuijinA vs KuijinB, followed by KuijinF vs KatyF.

The flavonol species were the most abundant among differential flavonoid species in the nine groups (**Figure 3A**). There were 20 differentially accumulated flavonols in the KuijinA *vs* KuijinB and KuijinF *vs* KatyF comparisons, which was more than in the other comparisons (**Figure 3A**). The differentially accumulated flavonols among the samples were identified by analyzing the relative flavonol contents. The KatyA *vs* KatyB, KatyA *vs* KatyF, KatyB *vs* KatyF, KuijinA *vs* KuijinB, KuijinA *vs* KuijinF, KuijinB *vs* KuijinF, KuijinA *vs* KatyA, KuijinB *vs* KatyB, and KuijinF *vs* KatyF comparisons revealed 1, 12, 11, 13, 11, 2, 14, 6, and 19 flavonols with decreasing contents, respectively (**Figure 3B**, **Table S3**). Although there are more different accumulate flavonols between KuijinA vs KatyA and KuijinF vs KatyF, ‘KatyA’ shows a greater decrease in flavonol content compared to its increase, while ‘KuijinF’ has more flavonols decreasing than increasing compared to’KuijinA’, indicating that ‘KuijinA’ has a higher flavonol content than both ‘KatyA’ and ‘KuijinF’.

Moreover, the following 10 flavonols accumulated more in the ‘KuijinA’ than in the other samples: quercetin 7-O-rutinoside (pmb0711), quercetin O-acetylhexoside (pmb3026), quercetin 3-O-rutinoside (pme0202), quercetin 3-alpha-L-arabinofuranoside (pme0361), kaempferol 3-O-rutinoside (pme0369), kaempferol 3-O-robinobioside (pme1606), quercetin 4′-O-glucoside (pme3129), quercetin 3-O-glucoside (pme3211), kaempferol 3-O-galactoside(pme3268), and quercetin 7-O-β-D-glucuronide (pme3442)(**Figure 3C**). These might be the key metabolites influencing the total flavonol content in the ‘KuijinA’.

### Ten flavonols contribute to the high flavonoid content in ‘KuijinA’ fruits

The flavonoid content of ‘Kuijin’ in its young fruit stage is higher than other stages, with the greatest variety of flavanols. This is mainly due to the presence of 10 types of flavanols. (**Figure 3A**). The relative contents of 10 flavonols were significantly higher in KuijinA than in the other samples (**Figure 3C**), including quercetin 3-O-rutinoside (rutin), kaempferol 3-O-rutinoside (nicotiflorin), and kaempferol 3-O-robinobioside (biorobin) (**Figure 3C**). Accordingly, these flavonols in apricot fruits are glycosylated at 3-O.

### Fruit transcriptome profiles in three developmental stages

The transcriptomes of the ‘Katy’ and ‘Kuijin’ fruits in three developmental stages were analyzed. The transcriptome sequencing data for 18 apricot samples, the sample correlation heat map (**Figure S2**), and the PCA plot (**Figure S3**) revealed biological replicates of the same species are grouped together. A total of 138.84 Gb clean data were obtained, with 108 Gb high-quality reads. The proportion of Q20 bases exceeded 96%, whereas the proportion of Q30 bases was between 89.15% and 91.57%. The GC content was greater than 45% (**Table S4**).

### Screening for differentially expressed genes related to flavonol synthesis

Different numbers of DEGs were detected in nine pairs of comparisons, including 990 for KatyA vs KatyB, 8,004 for KatyA vs KatyF, 5557 for KatyB vs KatyF, 3952 for KuijinA vs KuijinB, 9023 for KuijinA vs KuijinF, 6543 for KuijinB vs KuijinF, 4005 for KuijinA vs KatyA, 1105 for KuijinB vs KatyB, and 2299 for KuijinF vs KatyF (**Table S5**).

**Figure 4:**
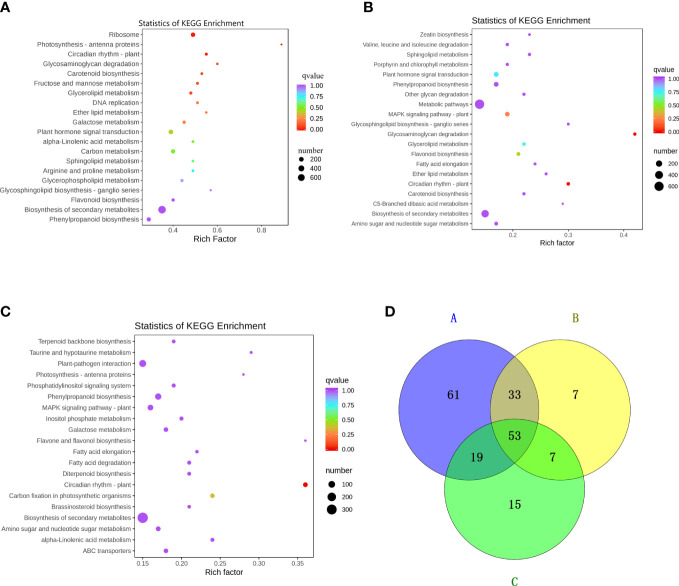
Preliminary analysis of transcriptome data. KEGG enrichment analysis of DEGs between the comparison groups **(A)** KuijinA *vs* KuijinF **(B)** KuijinA *vs* KuijinB **(C)** KuijinA *vs* KatyA. Each bubble in the plot represents a metabolic pathway. A larger bubble size indicates a larger impact factor. Darker bubble colors represent a higher degree of enrichment. **(D)** The number of common DEGs in KuijinA *vs* KuijinF (A represent it), KuijinA vs KuijinB (B represent it) and KuijinA vs KatyA (C represent it) (|log2Fold Change| ≥ 1, FDR< 0.05).

The grouping with significant differences in flavonol content consists of three pairs: KuijinA vs KuijinF, KuijinA vs KuijinB, and KuijinA vs KatyA (**Figure 2C**). KEGG enrichment analysis revealed that phenylpropanoid biosynthesis, flavonoid biosynthesis, and flavonol biosynthesis were enriched in three comparison groups: KuijinA vs KuijinF, KuijinA vs KuijinB, and KuijinA vs KatyA(**Figures 4A–C**). There were 166, 100, and 94 differentially expressed genes (DEGs) related to flavonols in the three comparison groups, respectively (**Figure 4D**). A Venn diagram analysis was performed on the differentially expressed genes of the three groups, which revealed that there were 53 commonly DEGs (**Figure 4D**). Among 53 DEGs, three structural genes strongly correlated to the biosynthesis of flavonols were identified (Pearson correlation coefficients > 0.8, p value< 0.05), including *PARG09190*, *PARG15135*, *PARG17939*. According to the analysis of gene function and RNA-Seq, three structural genes were positively correlated to the flavonols content (**Table S6**).

**Figure 5:**
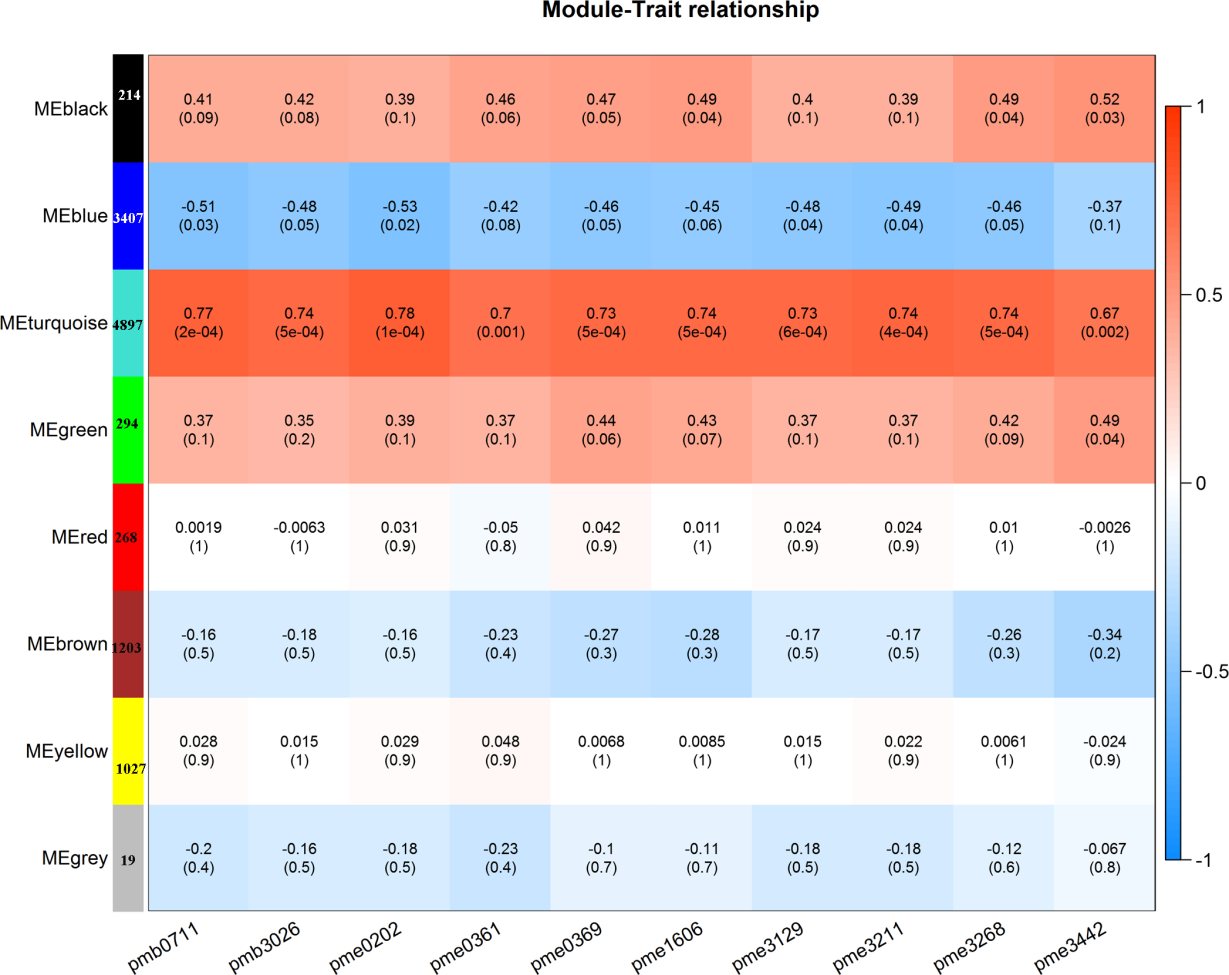
WGCNA-based screening for hub genes related to flavonol biosynthesis.

To gain further insight into the regulation of flavonols biosynthesis, we carried out WGCNA to investigate the co-expression gene modules and the critical modules involved in flavonols biosynthesis. A total of 8 co-expression modules were identified according to their expression patterns (**Figure 5**). The correlation between the gene matrix of different modules and the flavonols content was analyzed, and the correlation and corresponding e-value were presented in a digital form in the grid where each module and trait intersect (**Figure 5**). According to the ‘module character’ correlation analysis, the turquoise module showed a significant positive correlation with 10 flavonols. There were 4897 genes in this module.

**Figure 6:**
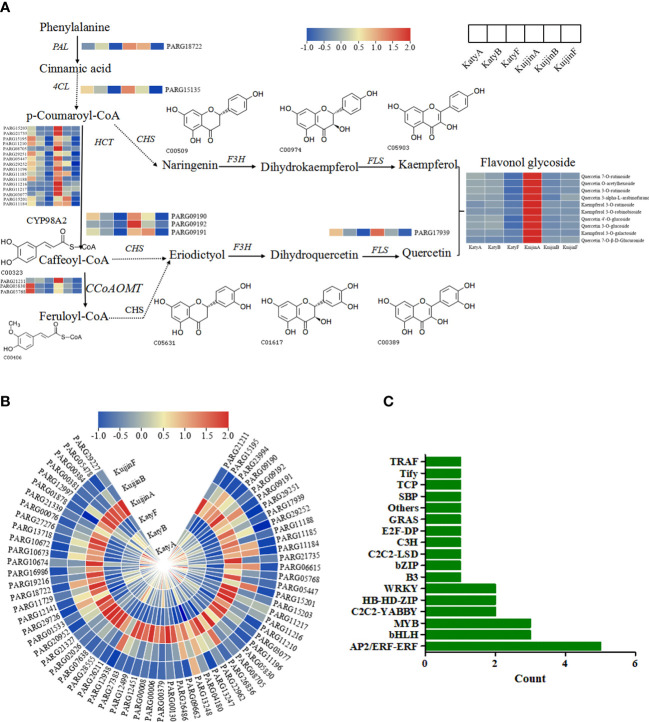
**(A)** Analysis of the DEGs in the flavonol biosynthesis pathway. **(B)** Heat map analysis of 66 gene expression levels using TBtools. **(C)** Bar Graph of 28 Transcription Factors. Count values increase as the number of transcription factors for that gene increases.

Among the 4897 genes in the turquoise module, 66 structural genes were identified involved in phenylpropanoid biosynthesis, flavonoid biosynthesis, and flavonol biosynthesis pathways. To generate the regulatory network associated with flavonols biosynthesis, we constructed the flavonol metabolic pathway in **Figure 6A**, and examined the structural genes involved in flavonols biosynthesis. Interestingly, 66 DEGs including *PARG09190*, *PARG15135*, *PARG17939*, were recognized both in the biosynthesis of flavonols in the turquoise module, whose expression was highly correlated with the 10 flavonols content (**Figure 6B**), suggested that these DEGs correspond to the regulation of flavonols biosynthesis.

**Figure 7:**
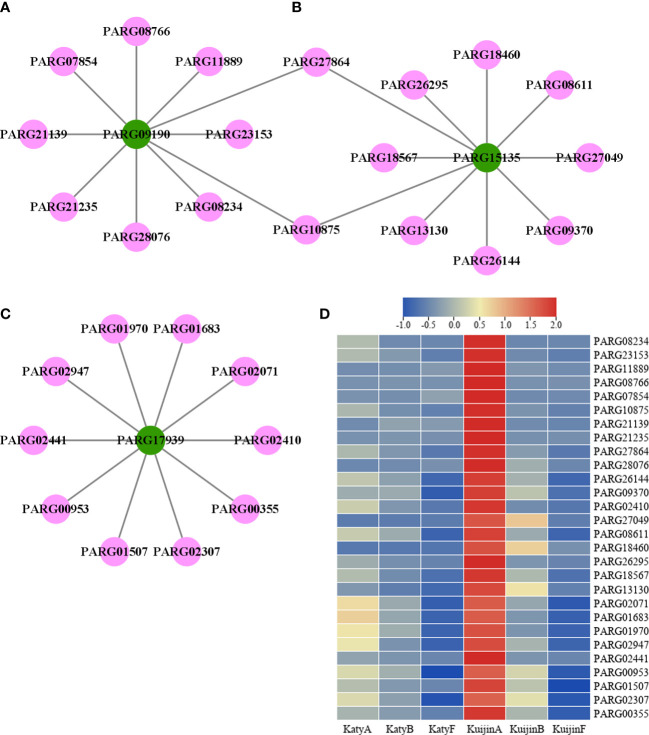
Co-expression network of transcription factors and structural genes potentially involved in the flavonols metabolism. **(A)** The network of 10 transcription factors and PARG09190. Green circles represent structural genes, and pink circles represent TFs. **(B)** The network of 10 transcription factors and PARG15135 **(C)** The network of 10 transcription factors and PARG17939. **(D)** Heatmap of 28 transcription factors.

According to weight value, the top ten transcription factors associated with each of the three structural genes were selected from 4897 genes in the turquoise modules. A total of 28 transcription factors were screened (**Figure 6C**). The structural genes and transcription factors were organized into a connection network using Cytoscape software. In the turquoise module, the top 10 transcription factors (TFs) associated with *PARG09190* were *PARG08234* (AP2/ERF-ERF), *PARG23153* (C2C2-LSD), *PARG11889* (Tify), *PARG08766* (AP2/ERF-ERF), *PARG07854* (MYB), *PARG10875* (TCP), *PARG21139* (MYB), *PARG21235* (AP2/ERF-ERF), *PARG27864* (AP2/ERF-ERF) and *PARG28076* (C2C2-YABBY)(**Figure 7A**). The top 10 TFs and transcriptional regulatory factor (TR) associated with *PARG15135* were *PARG26144* (bHLH), *PARG09370* (C3H), *PARG27864* (AP2/ERF-ERF), *PARG10875* (TCP), *PARG27049* (B3), *PARG08611* (HB-HD-ZIP), *PARG18460* (TRAF), *PARG26295* (Others), *PARG18567* (bHLH), *PARG13130* (MYB)(**Figure 7B**). The top 10 TFs associated with *PARG17939* were *PARG02410* (E2F-DP), *PARG02071* (GRAS), *PARG01683* (WRKY), *PARG01970* (AP2/ERF-ERF), *PARG02947* (bHLH), *PARG02441* (SBP), *PARG00953* (HB-HD-ZIP), *PARG01507* (C2C2-YABBY), *PARG02307* (WRKY), *PARG00355* (bZIP)(**Figure 7C**). Interestingly, two common transcription factors, including PARG27864 and PARG10875 associated with *PARG09190* and *PARG15135* in the module, whose expression was highly correlated with the 10 flavonols content (**Figure 7D**).

### Hub gene screening and visualization

To identify potential genes related to the metabolism of 10 flavonol compounds, candidate genes and regulators were analyzed using Cytoscape software. Three structural genes were identified: CYP98A2 (*PARG09190*), 4CL (*PARG15135*), and FLS (*PARG17939*). Based on the weight value, 28 transcription factors are associated with 3 structural genes. Two of the transcription factors are not only associated with *PARG09190* but also with *PARG15135*, indicating their critical importance in the flavonols biosynthesis.

### Quantitative PCR-based confirmation of gene expression

To validate the RNA-seq (i.e., transcriptome sequencing) data and the contribution of specific genes to flavonol synthesis, degradation, and transport, we selected 12 genes for a qPCR analysis. The qPCR data for seven structural genes and five transcription factor genes were consistent with the FPKM values derived from the RNA-seq data (**Figure S4**).

## Discussion

### Flavonols detected in ‘Katy’ and ‘Kuijin’ apricot fruits

Metabolites are the final products of cellular regulatory processes, and their levels vary in response to genetic and environmental changes (Fiehn, 2002; García-Gómez et al., 2021). Flavonols are major flavonoids with biological functions (Barbehenn and Constabel, 2011). For example, they participate in auxin transport and responses to oxidative stress (Agati et al., 2012; Brunetti et al., 2013). Previous studies on apricot fruits primarily focused on fruit flavors (e.g., sweetness due to sugars and acidity) and flesh colors (Zhang et al., 2019; Zhou et al., 2020).

In the current study, we observed that for ‘Katy’ and ‘Kuijin’, flavonol glycosides were more abundant in young fruits than in mature fruits (**Figure 2A**). We applied a comprehensive targeted metabolomics approach to analyze the secondary metabolites of ‘Katy’ and ‘Kuijin’ fruits during three developmental stages. A total of 572 metabolites were detected, and the changes in their contents in developing fruits were explored (**Figure 2C**).

Our analyses showed that the flavonoid content was highest in KuijinA, with flavonol glycosides accounting for a considerable proportion of the accumulated flavonoids (**Figure 2C**). According to the molecular structure of the flavonols, eight glycosides were detected, including three monoglycosides (glucose and galactoside), four polyglycosides (rhamnoside, rutinoside, neohesperidoside, and robinobioside), and one glucuronide (**Figure 3C**). These results suggested that the flavonols were mainly glycosylated at 3-O, 5-O, 7-O, and the C-position, which is consistent with the findings of earlier studies (Barreca et al., 2011; Xiao et al., 2014).

Glycosidases catalyze the production of flavonol glycosides, including monoglycosides (e.g., glucosides, galactosides, and rhamnosides), diglycosides [e.g., rutoside (6-O-α-L-rhamnose-D-glucose) and neohesperidin (2-O-α-L-rhamnose-D-glucose)], and polyglycosides (Xiao, 2017; Peterson and Dwyer, 1998).

### Key structural genes responsible for flavonol synthesis in ‘Katy’ and ‘Kuijin’ apricot fruits

The mechanisms mediating flavonoid synthesis have been elucidated in many plant species. There has recently been significant progress in the research on the flavonoid synthesis in fruit crops, including grape(Boss et al., 1996), strawberry(Almeida et al, 2007), apple(Takos et al, 2006; Vimolmangkang et al., 2013), and pear(Fischer et al, 2007). The key enzymes involved in flavonol synthesis include flavonol synthase (FLS)(Martens et al., 2002), which belongs to the 2-oxoglutarate-dependent dioxygenase superfamily (Park et al., 2019; García-Gómez et al, 2021). Transgenic tobacco (*Nicotiana tabacum*) plants expressing *OsFLS* produce pale pink or white flowers; their petals contain significantly more and less kaempferol-3-O-rutinoside and anthocyanins, respectively, than the wild-type petals (Park et al., 2019). Considering the differences between KuijinA and the other examined samples in terms of their flavonol contents, three DEGs may be critical for flavonol accumulation of which *PaFLS (PARG17939), CYP98A2(PARG09190), Pa4CL(PARG15135)* may be essential for flavonol production.

In *Arabidopsis thaliana*, UGT73C6 catalyzes the transfer of glucose from UDP-glucose to the 7-OH position of kaempferol-3-O-rhamnoside and quercetin-3-O-rhamnoside(Jones et al., 2003). In the current study, *PaUGT73C* (*PARG22962*) expression was highly correlated with the kaempferol 3-O-rutinoside (nicotiflorin) content. An earlier study confirmed that AtUGT74F1 glycosylates quercetin at the 4′- and 7-hydrogen sites (Lim et al., 2004). In our study, *PaUGT74F2* (*PARG29757*) expression was correlated with the quercetin 7-O-rutinoside content.

### Transcription factors responsible for flavonol synthesis in ‘Katy’ and ‘Kuijin’ apricot fruits

The flavonoid pathway is regulated by a highly conserved MYB–bHLH–WD transcriptional complex (Stracke et al., 2001; Wang et al., 2011; Schaart et al., 2013; Xu et al., 2014; Xu and Huang, 2014). In grapes, VvMYBF1 regulates VvFLS1 expression, thereby modulating flavonol biosynthesis (Czemmel et al., 2009). Moreover, AtMYB11, AtMYB12, and AtMYB111 promote flavonol synthesis in A. thaliana (Stracke et al., 2007), while PbMYB12b positively regulates flavonol production in ‘Red Zaosu’ pear fruit (Zhai et al., 2019).

VvMYB86 may negatively regulate anthocyanin synthesis, or positively regulate flavonol synthesis (Sun, 2020). PARG02716 and AtMYB86 belong to the SG13 subfamily. Vimolmangkang et al (2013) overexpression of apple SG4 MYB protein was found MdMYB3 promotes the synthesis of anthocyanin and flavonol in tobacco. PARG14380 and AtMYB6 belong to the SG4 subfamily. Additionally, the AtMYB11, AtMYB12, and AtMYB111 genes reported by Stracke et al. also belong to the SG7 subfamily (Stracke et al., 2007). The three MYB TFs (PARG07517, PARG07518, and PARG27682) were putatived to have similar expression. AtMYB12 was originally identified as a flavonol-specific transcriptional activator in Arabidopsis thaliana, and this has been confirmed by ectopic expression in tobacco. AtMYB12 is able to induce the expression of additional target genes in tobacco, leading to the accumulation of very high levels of flavonols (Zhai et al., 2019).

Bovy et al (2002) expressed the maize transcription factor genes *LC* and *C1* (MYC-type) in the fruits of genetically modified tomato plants to increase flavonoid levels; these fruits accumulated relatively large amounts of kaempferol (flavonol) in their flesh. In our study, the transcription factor genes associated with flavonol accumulation included 12 belonging to the *bHLH* family, of which the highest kwith value is bHLH106 (*PARG26144)* (**Table S7**).

*PARG27864* was included in the turquoise module following the WGCNA (**Table S7**). The AP2/ERF transcription factors reportedly regulate flavonol synthesis in apple (Bovy et al., 2002). AtERF11 - Facilitates flavonol synthesis in celery by directly binding to the *PAL* gene promoter(Xu et al., 2014; Xu and Huang, 2014). Pti4 - Promotes the expression of several key genes in the flavonol synthesis pathway in tomato (Li et al., 2006). ERF98 - Affects fruit quality and alkaloid content in pomegranate by regulating the expression of genes involved in flavonol synthesis pathway (Zhang et al., 2018).

## Conclusion

In this study, metabolome and transcriptome analyses were performed to identify key flavonols and the genes associated with their biosynthesis in apricot fruits. A total of 572 metabolites were detected in ‘Katy’ and ‘Kuijin’ pulp, including 111 flavonoids. The higher flavonol content in Kuijin during its young fruit stage is mainly due to 10 types of flavonols. Three pairs of significant differences in flavonol content were identified: KuijinA vs KuijinF, KuijinA vs KuijinB, and KuijinA vs KatyA. From these three comparison groups, three structural genes were strongly correlated with the levels of 10 types of flavonols (Pearson correlation coefficients > 0.8, p value < 0.05), including *PARG09190*, *PARG1513*5, and *PARG17939*. We carried out WGCNA to investigate the co-expression gene modules and the critical modules involved in flavonols biosynthesis. A total of 8 co-expression modules were identified according to their expression patterns. The turquoise module showed a significant positive correlation with 10 flavonols. There were 4897 genes in this module. Out of 4897 genes, 28 transcription factors are associated with 3 structural genes based on weight value. Two of the transcription factors are not only associated with *PARG09190* but also with *PARG15135*, indicating their critical importance in the flavonols biosynthesis. These two TFs are *PARG27864* and *PARG10875*.

## Data availability statement

The datasets presented in this study can be found in online repositories. The names of the repository/repositories and accession number(s) can be found in the article/**Supplementary Material**. However, I found the following words: Global list: FigShare, RNASeq, RNA-seq. Please check if the data have to be deposited in a public repository.

## Author contributions

XH, JW, and XX conceived and designed the experiments. XH, PN, GW performed the experiments. XH and FD analyzed the data. XH wrote the manuscript. All the authors read and approved the manuscript.

## Supplementary material

Supplementary Figure 1 3D diagram of PCA of all grouped samples in metabolome analysis.

Supplementary Figure 2 Statistical chart of correlation between biological duplicate samples.

Supplementary Figure 3 Principal component analysis diagram of samples in transcriptome analysis.

Supplementary Figure 4 Biosynthetic genes and transcription factors regulating flavonol synthesis used for qRT-PCR validation.

Supplementary Table 1 SPSS analysis of total flavonoids in ‘Katy’ and ‘Kuijin’ apricotat three periods.

Supplementary Table 2 All sample metabolome data.

Supplementary Table 3 Type and content of flavonols in 9 groups.

Supplementary Table 4 Clean reads data.

Supplementary Table 5 Summary of differential gene data in 9 groups.

Supplementary Table 6 Correlation analysis between 53 differentially expressed genes and 10 flavonol compounds.

Supplementary Table 7 TFs in turquoise module from WGCNA data.

Supplementary Table 8 Primer sequences used in qRT-PCR.

Supplementary Table 9 Genes in turquoise module from WGCNA data.

Supplementary Table 10 Differentially expressed genes (DEGs) shown in **Figure 6**.
