# Supplementary figures and images for: Transcriptome and metabolome analysis of flavonol synthesis in apricot fruits
Source: Front Plant Sci. 2023 Jun 14;14:1187551. doi: 10.3389/fpls.2023.1187551 (PMC10303810; doi:10.3389/fpls.2023.1187551)

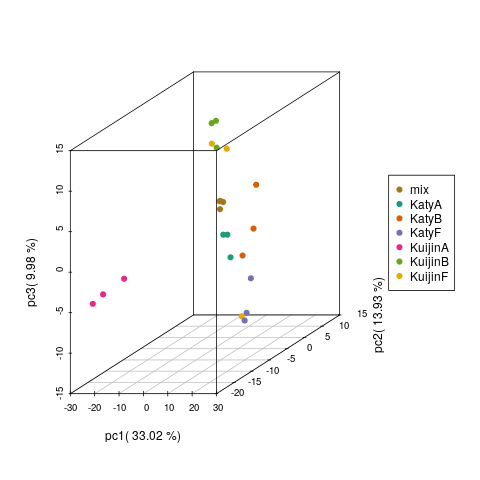

Supplement: Supplementary Figure 1 — 3D diagram of PCA of all grouped samples in metabolome analysis. [file Image_1.tif]

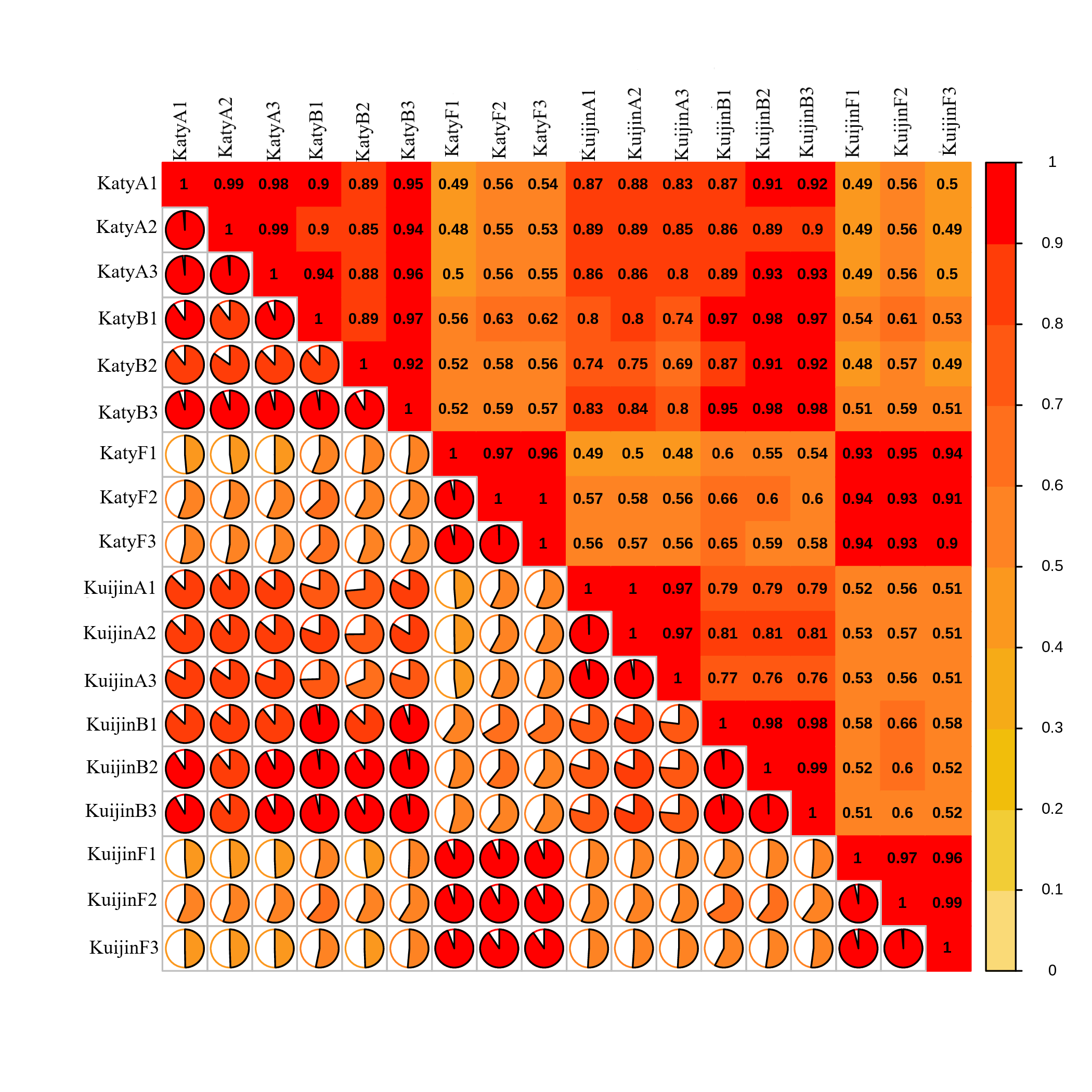

Supplement: Supplementary Figure 2 — Statistical chart of correlation between biological duplicate samples. [file Image_2.png]

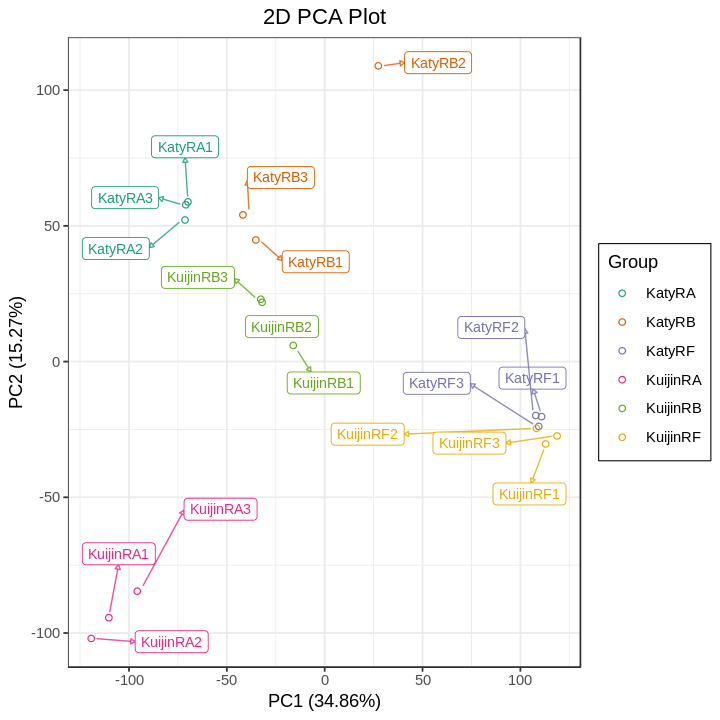

Supplement: Supplementary Figure 3 — Principal component analysis diagram of samples in transcriptome analysis. [file Image_3.png]

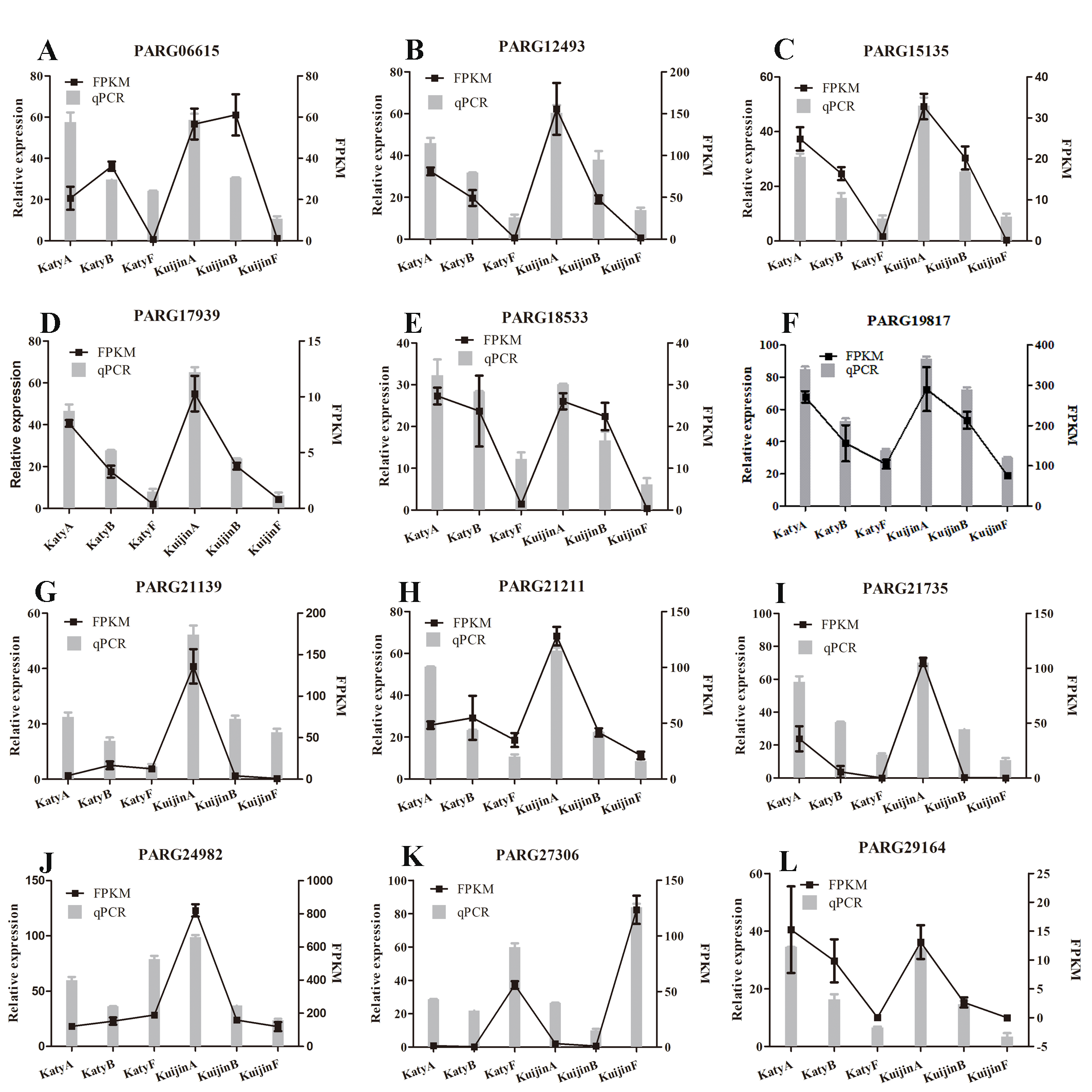

Supplement: Supplementary Figure 4 — Biosynthetic genes and transcription factors regulating flavonol synthesis used for qRT-PCR validation. [file Image_4.png]
